# Varietal Authentication of Extra Virgin Olive Oils by Triacylglycerols and Volatiles Analysis

**DOI:** 10.3390/foods8020058

**Published:** 2019-02-05

**Authors:** Francesca Blasi, Luna Pollini, Lina Cossignani

**Affiliations:** University of Perugia, Department of Pharmaceutical Sciences, Section of Food Science and Nutrition, Via San Costanzo, 06126 Perugia, Italy; francesca.blasi@unipg.it (F.B.); luna.pollini@studenti.unipg.it (L.P.)

**Keywords:** authenticity, olive oil, cultivars, quality, triacylglycerols, volatiles, chemometrics

## Abstract

In recent years, there is an increasing interest in high-quality extra virgin olive oils (EVOOs) produced from local cultivars. They have particular chemical/organoleptic characteristics and are frequently subjected to fraud, whereby the control of quality requires a powerful varietal check. In the present research, triacylglycerols (TAGs) and volatiles have been studied as chemical markers for the authentication of EVOO samples from four Italian varieties of *Olea europea* (Dolce Agogia, Frantoio, Leccino, and Moraiolo). The monocultivar EVOO samples have been subjected to a chemical–enzymatic chromatographic method in order to perform a stereospecific analysis, an important procedure for the characterization of TAG of food products. The results, combined with chemometric analysis (linear discriminant analysis, LDA), were elaborated in order to classify Italian EVOO monocultivar samples. In accordance with the total and intrapositional fatty acid (FA) composition of TAG fraction, the results were allowed to carry out a varietal discrimination. In addition, volatile compounds were also determined by solid-phase micro-extraction gas chromatography-mass spectrometry analysis. All EVOO samples were correctly classified when TAG stereospecific data and volatile results were elaborated by the LDA procedure, even if volatile compounds showed a higher discriminant power.

## 1. Introduction

Extra virgin olive oil (EVOO), obtained from the fruit of *Olea europea* L. using only physical or mechanical methods, is a product of great importance because of its exclusive chemical-nutritional characteristics, and health properties. EVOO is one of the key ingredients in the Mediterranean diet [1].

The production of olive oils from monovarietal olives is carried out to produce oils with particular chemical composition and unique organoleptic properties, which depend on cultivar, geographic origin, and pedoclimatic conditions [2].

The check of the cultivars used to obtain an olive oil may contribute to highlight the oil origin. This aspect may have commercial interest in the case of monovarietal high-quality EVOO with typical marks (protected designation of origin-PDO, protected geographical indication-PGI, traditional specialty guaranteed-TSG), because these oils have high commercial value and may be adulterated by lower quality oils, using anonymous or less expensive cultivars [3].

There is an increasing need for developing appropriate methodologies in order to guarantee food traceability [4], as well as to identify geographical origin [5] or cultivar [6]. 

For EVOO traceability, several analytical approaches, from chromatographic to nondestructive spectroscopy [7,8] have been reported, together with DNA based methods or electrochemical devices [9,10]. Studies of authenticity have been reported for the classification of olive oils according to their botanical or geographical origin, based on determination of fatty acid (FA) profile or minor constituents, as phytosterols, phenols, or volatiles [11,12,13]. In addition, the classification was performed using a simultaneous combination of two or more components; for example nuclear magnetic resonance (NMR) [14], Fourier Transform Infra-Red spectroscopy [15], and stable isotopic techniques [16,17] have been used.

The differentiation of EVOO samples according to variety and geographical origin has been recently addressed by comprehensive two-dimensional gas chromatography [18] and by ultra-high-pressure liquid chromatography (UHPLC) coupled to an electrospray quadrupole–time-of-flight hybrid mass spectrometer (ESI/QTOF-MS) [19,20]. Omics-based massive molecular tools can help to circumvent limitations of traditional methodologies, and therefore genomics, proteomics and metabolomics-based methods are being developed for the authentication of a wide range of food commodities [21].

In this research, the characterization of olive oil varieties was performed initially by triacylglycerol (TAG) stereospecific analysis. Afterwards, volatile analysis by solid-phase microextraction gas chromatography-mass spectrometry (SPME-GC–MS) was carried out.

The objectives of this paper have been: (i) To study the TAG fraction of monocultivar EVOO, for total and positional FA compositions, resulting from the specificity of biosynthetic enzymes and correlated to the nutritional aspects; (ii) to obtain the qualitative and quantitative profile of EVOO volatile fraction, also depending on the enzymatic pool, directly related to genetic characteristics; and (iii) to investigate and compare the potential of stereospecific analysis of TAG and of volatile profile, combined with chemometric data analysis, to classify four monovarietal Italian EVOO (Dolce Agogia, Frantoio, Leccino, and Moraiolo) on the basis of varietal origin.

## 2. Materials and Methods

### 2.1. Materials and Chemicals

Acetone, diethyl ether, hydrochloric acid, formic acid, methanol, and petroleum ether were obtained from J.T. Baker B.V. (Deventer, the Netherlands). Anhydrous sodium sulfate, chloroform, ethanol, hexane, and potassium hydroxide were purchased from Carlo Erba Reagents (Milan, Italy). Deionized water was from a Milli-Q SP Reagent Water System (Bedford, MA, USA). Supelco™ 37 component fatty acid methyl esters (FAME) mix (catalog n° 47885-U), containing the methyl esters of 37 fatty acids (the FA contents ranged between 2% and 4%, while the palmitic acid methyl ester was 6%), was bought from Supelco (Bellefonte, PA, USA). Divinylbenzene/carboxen/polydimethylsiloxane (DVB/CAR/PDMS) fiber, lipase from porcine pancreas (EC 3.1.1.3), and *sn*-1,2-diacylglycerol kinase from *Escherichia coli* (DAGK; EC 2.7.1.107) were acquired from Sigma-Aldrich (St. Louis, MO, USA). 

### 2.2. EVOO Samples

Sixteen EVOO samples from four different *O. European* cultivars (Dolce Agogia, Frantoio, Leccino, and Moraiolo), typical of Central Italy, were analyzed. Monovarietal bottled EVOO samples were purchased in 2016 from local producers, which guaranteed their origin and cultivar. The monovarietal EVOO samples were stored in the dark at 8 °C.

### 2.3. Purification of TAG Fraction from EVOO Samples

The TAG fraction was isolated from monovarietal EVOO samples by thin layer chromatography as reported in a previous paper [22].

### 2.4. Stereospecific Analysis of TAG Fraction from EVOO Samples

The stereospecific analysis procedure [23] was performed on purified TAG of EVOO samples, and the following steps were carried out:

(a) Hydrolysis by pancreatic lipase to obtain *sn*-2-monoacylglycerols and then the FA percent positional composition of TAG *sn*-2 position;

(b) TAG deacylation by Grignard reagent to obtain *sn*-1,3/*sn*-1,2(2,3)-diacylglycerols, followed by the DAGK enzymatic reaction in order to obtain the *sn*-1,2-phosphatidic acids and then the FA percent positional composition of TAG *sn*-1 and *sn*-2 positions.

### 2.5. Preparation of Methyl Esters of Constituent Fatty Acids and Analysis

Fatty acid methyl esters (FAME) were prepared by transesterification, as previously reported [24] and analyzed by high resolution gas chromatography (HRGC). A DANI 1000DPC gas-chromatograph (Norwalk, CT, USA) provided with a split–splitless injector and a flame ionization detector (FID) was used. A fused silica capillary column, named CP-Select CB for FAME (50 m × 0.25 mm i.d., 0.25 μm f.t.; Varian, Superchrom, Milan, Italy), was used for the chromatographic separation. The injector and detector temperature was 250 °C. The initial oven temperature, 180 °C, was held for 6 min, raised at 3 °C/min to 250 °C, and finally maintained for 10 min. Carrier gas was helium with flow rate of 1 mL/min; the injection volume was 1 µL with a split ratio of 1:70. To identify the FA, the standard mixture containing 37 FAME was used. The percentage of each FA was calculated using the peak area of the samples. The chromatograms were acquired and processed using Clarity integration software (DataApex Ltd., Prague, Czech Republic). The data were normalized considering only the main reported FA (% mol mean values ≥0.1).

### 2.6. Analysis of Volatile Fraction

Volatiles have been analyzed by SPME-GC–MS as reported in a previous paper [25]. The heated samples were placed in a 25 °C water bath and the DVB/CAR/PDMS fiber, previously cleaned for 15 min at 250 °C, was exposed to the sample headspace for 15 min. 

Volatile compounds were analyzed with a Hewlett-Packard 5890 series II gas chromatograph (Palo Alto, CA, USA) equipped with a split–splitless injector, an Econo-Cap EC-5 capillary column (30 m × 0.25 mm i.d., 0.25μm f.t.; Alltech, Milan, Italy), and a 5971A quadrupole MS detector (Palo Alto, CA, USA).

Volatiles were desorbed from the SPME fiber at 250 °C for 5 min into the injector port, in splitless mode. The oven initial temperature, 35 °C, was maintained for 5 min, then the temperature was raised at 3 °C/min to 230 °C, and finally maintained for 5 min.

The mass spectrometer operated in electron impact mode with electron energy of 70 eV, and scanned, in full scan acquisition mode, in the mass range 35–500 m/z at 1.2 scans/s. The temperatures of interface and ion source were 280 and 180 °C, respectively. Data were collected by HP G1030 MS ChemStation (Hewlett-Packard). Compounds were identified by comparing their mass spectra with those reported in Wiley138 mass spectral library and using the linear retention indexes from literature [26,27]. A semi-quantitative analysis was performed.

### 2.7. Statistical Analysis

All analytical determinations were performed in triplicate, and the reported results (FA compositional data and volatile data) were expressed as mean values and standard deviation (±SD). Data were processed and edited with Microsoft Excel 2016 (Microsoft, Redmond, WA, USA). LDA, used for the differentiation and classification of samples, was performed by SPSS Professional Statistics software (version 9.0 for Windows, IBM, Armonk, NY, USA).

## 3. Results and Discussion

### 3.1. Triacylglycerol Fraction

The results of this research confirm that EVOOs show a high percentage (from 76.2% of Leccino to 78.0% of Dolce Agogia) of oleic acid, a medium content of palmitic (from 12.3% of Dolce Agogia to 13.2% of Leccino) and stearic (about 2%) acids, a good percentage of linoleic acid (from 6.0% of Dolce Agogia to 7.1% of Frantoio), and a low content of α-linolenic (about 0.7%) acid. Table 1 shows the data of the acidic compositions of the total TAG percent composition of monovarietal EVOO samples.

The linearity parameters, evaluated in the range 12.5–200 µg/mL of the considered FAME, have been calculated together with their limits of detection (LOD) and limits of quantification (LOQ), according to the statistical method, reported in Federal Register [28]. The coefficients of correlations for the considered FAME were always greater than 0.9969. The LOD values change from 10 ng/mL of α-linolenic acid to 20 ng/mL of palmitic acid. The LOQ values change from 30 ng/mL of α-linolenic acid to 66 ng/mL of palmitic acid.

Similar FA composition was obtained from other analyzed EVOO samples [20]. In a previous work, the leaves of the same olive tree varieties (Dolce Agogia, Frantoio, Leccino, and Moraiolo) have been studied to evaluate the seasonal variations of antioxidant compounds, and significant differences in hydroxytyrosol and oleuropein contents among the cultivars have been observed [29].

Some slight differences (*p* > 0.05) of total FA percent compositions were observed. Considering, for example, the Dolce Agogia and Leccino varieties, a higher percent content of oleic acid and a lower percent content of linoleic acid, the first in respect to the second cultivar, were found. Recently, the effects of different cultivar (Arbequina, Leccino, Maurino and Moraiolo) on the qualitative and quantitative profile of EVOOs have been studied by determining the profiles of acylglycerides, sterols, phenolics, hydrocarbons, pigments, and volatile components [8]. Regarding FA composition (palmitic, oleic, linoleic acids), as a function of cultivar and harvest date (2015), significantly different data were obtained in respect to those reported in Table 1.

It is known that the structure of glycerol backbone of TAG fraction influences the physicochemical, physiological, and nutritional properties of lipids. A deeper knowledge of these aspects can be useful to determine the geographical origin, species, varieties of a fat, as well as to detect food fraud, and to predict nutritional value. The dietary fats are absorbed mainly as *sn*-2-MAG and also as free FA, produced by lipase hydrolysis. They are re-esterified as TAG and incorporated into chylomicrons. In this way, the absorbed TAG molecules maintain the FA in the *sn*-2 position as in the dietary TAG, whereas the FA in the *sn*-1 and *sn*-3 positions are randomized and partly substituted by endogenous FA. Consequentially, the positions esterified by FA in the glycerol backbone become important for physiological/nutritional reasons [30]. In addition, another interesting application of enzymatic-instrumental methods is the monitoring of the synthesis of structured lipids to obtain better healthy fats [31,32,33]. For example the production of TAG with interesting FA esterified in *sn*-2 position, with nutritional advantages due to their real bioavailability, or medium chain triglycerides useful for enteral nutrition.

Table 2 shows the data of the acidic percent compositions of the *sn*-1, *sn*-2, and *sn*-3 positions of the TAG. With regard to the intrapositional acidic compositions, some significant differences among the cultivars were observed (*p* < 0.05). For example, considering the acidic composition of the *sn*-1 position, a lesser incorporation of palmitic acid in the *sn*-1 position (15.4 vs. 18.6/17.5/17.6) in Dolce Agogia variety, was observed compared to Frantoio, Leccino, and Moraiolo. A lower content of α-linolenic acid in the *sn*-1 position was found in Dolce Agogia variety compared to Leccino and Moraiolo (*p* < 0.05). A minor incorporation of linoleic acid in the *sn*-2 position of TAG of Dolce Agogia variety was observed compared to Frantoio and Leccino (8.9 vs. 10.5/10.8). Moreover, lower percent content of palmitic acid in *sn*-2 position has been highlighted in Dolce Agogia variety, compared to Frantoio (*p* < 0.05). 

The data of the intrapositional compositions of the TAG could also be used to obtain all the TAG molecular species, including the isomeric and enantiomeric species in order to evaluate the differences in the content of individual species among the different cultivars. It is, however, important to emphasize that the procedure for TAG stereospecific analysis has the disadvantage of being laborious, time-consuming, and difficult to automate.

In this study, the little differences highlighted from stereospecific analysis data were better revealed applying a chemometric procedure as LDA, reported below.

### 3.2. Volatile Fraction

**Figure 1:**
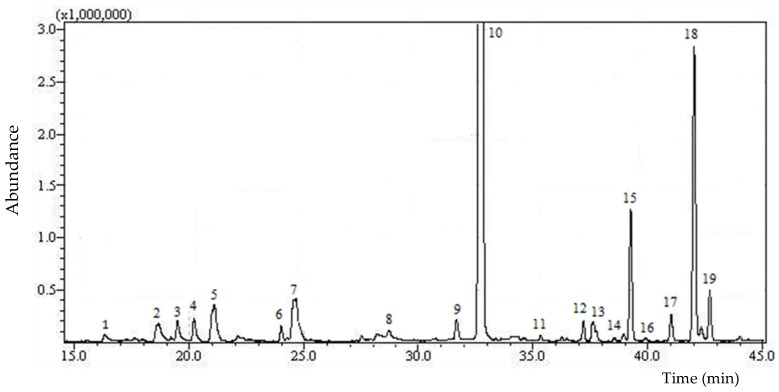
High-resolution gas chromatography mass-spectrometry (HRGC-MS) profile of volatile fraction of a monovarietal EVOO (Dolce Agogia) sample. Peak numbers correspond to the compounds listed in Table 3.

In this investigation SPME-GC–MS, a simple and effective analytical method, was performed to obtain qualitative and semi-quantitative profiles of volatiles from EVOO samples. Figure 1 shows the chromatographic profile of the volatile compounds of a monovarietal EVOO (Dolce Agogia) sample. 

The semi-quantitative results (% areas) of the considered EVOO samples are shown in Table 3. 

Some differences have been highlighted among the cultivars, both as regards the percent content of the *trans*-2-hexenal, the compound most represented in all cultivars, and other minor components. The *trans*-2-hexenal, which is formed from the α-linolenic acid by the action of the lipoxygenase enzymes, hydroperoxide lyase and isomerase, has been found in less amounts in the Moraiolo cultivar with respect to the Frantoio variety (*p* < 0.01), and also to Leccino and Dolce Agogia (*p* < 0.01). The content of this unsaturated aldehyde was also significantly different between Dolce Agogia - Frantoio (*p* <0.01) and Leccino (*p* < 0.05) varieties. Other observations regard the percent content of the *trans*-2-hexen-1-ol. This compound is obtained from the *trans*-2-hexenal by alcohol dehydrogenase activity, and its contents show an opposite trend with respect to its precursor (*trans*-2-hexenal). It was more represented in Moraiolo variety, followed by Dolce Agogia, Leccino and Frantoio. The differences between the cultivars are also enhanced if the relationship between the two compounds was considered. Based on the results obtained, it could be affirmed that the activity of the enzyme alcohol dehydrogenase is influenced by the variety.

EVOO cultivar discrimination by volatile analysis has been addressed by numerous other authors. In this regard, SPME-GC-MS technique has also been used to discriminate Italian monovarietal EVOO [34,35]. Moreover, in a recent paper several approaches for the varietal differentiation of monovarietal virgin olive oils are overviewed [36].

### 3.3. Discriminant Analysis

Previous papers have shown that TAG stereospecific analysis coupled with multivariate statistical data analysis was successfully used to characterize vegetable [22,23,37] and animal [38,39,40] foods. Generally, LDA is the best known and more widely used method to highlight differences between groups and to classify them. Moreover, it is known that LDA is an important parametric method useful to discriminate samples when the sample allocation is just known [41]. 

In this study, in order to classify and discriminate EVOO samples of different cultivars (Dolce Agogia, Frantoio, Leccino, and Moraiolo), multivariate parametric LDA technique was used. To better evaluate the influence of FA (total and intrapositional) compositions and volatile fraction in the classification of the oils, the results of the analytical determinations were elaborated by LDA. The statistical elaborations were performed considering the total and intrapositional FA percent compositions in TAG positions (*sn*-1, *sn*-2, and *sn*-3) of monovarietal EVOO samples, reported in Table 1 and Table 2, respectively. Afterwards, the statistical elaborations were performed considering the volatile percent compositions of monovarietal EVOO samples, reported in Table 3. 

The statistical elaboration of the results of the stereospecific analysis of the TAG provided interesting results for the characterization of oil samples obtained from different varieties of *O. europea*; the theory that the intrapositional TAG compositions represent a fingerprint of the most represented fraction of the oils is confirmed. In fact, these compositions depend on the specificity of the acyltransferase involved in the process of biosynthesis of the TAG, and are therefore species-specific [42].

The selection of the most significant variables was performed by stepwise analysis. Table 4 shows the canonical Fisher’s linear discriminant characteristics (eigenvalue, percentage of variance, and significance test) of the testing data (FA compositions and volatiles) from monovarietal EVOO samples. It can be emphasized that LDA performed on volatile data showed higher percentage of the variance explained (97.8 vs 54.8) and canonical correlation (1.000 vs 0.965) for the first discriminant function in respect to FA compositional data. The statistical significance of each discriminant function was also evaluated on the basis of the Wilks’ lambda factor; it showed that the first two functions for volatile data were significant (*p* < 0.05). In fact, Wilks’ lambda values of the first two discriminant functions (0.000) showed an optimal discriminant power of the model, with a better discriminant power of volatiles in respect to FA data. The significance values (0.000 for the first two functions) indicated that there was a highly significant difference between the group centroids for volatile data. 

**Figure 2:**
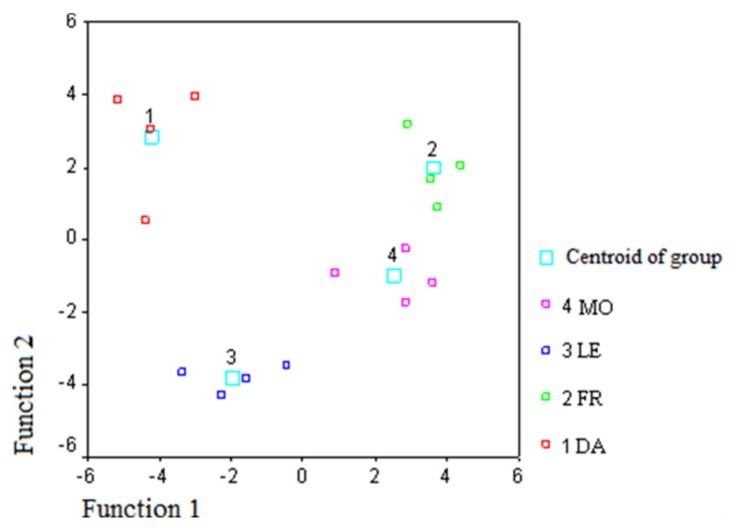
Discriminant function plot of the first two functions obtained using total and intrapositional FA percent composition of monovarietal EVOO samples (DA, Dolce Agogia; FR, Frantoio; LE, Leccino; MO, Moraiolo).

**Figure 3:**
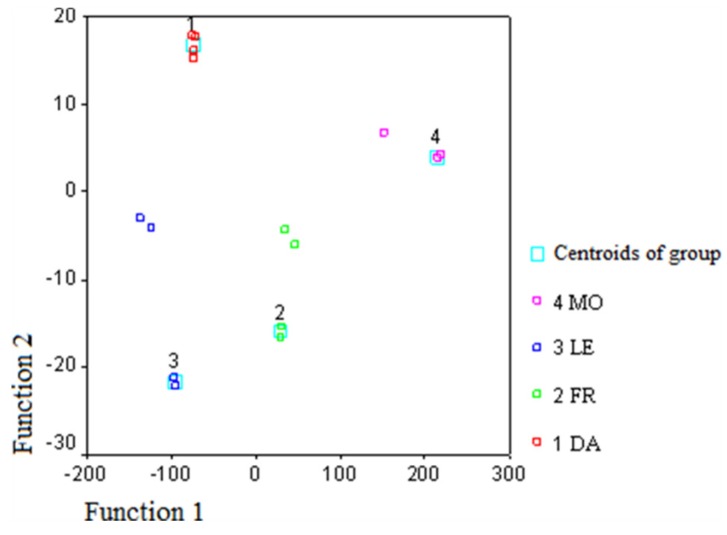
Discriminant function plot of the first two functions obtained using volatile percent composition of monovarietal EVOO samples (DA, Dolce Agogia; FR, Frantoio; LE, Leccino; MO, Moraiolo).

Table 5 shows the standardized canonical discriminant function coefficients. According to standardized coefficients, oleic and linoleic acids for FA had the greatest impact on the discrimination for functions 1 and 2. As regards the volatiles, ethanol and 1-penten-3-one had the greatest impact on the discrimination for function 1. The values showed the impact of each variable on the discriminant function after “standardizing”, putting each variable on the same platform. Figure 2 shows the plot of the first two discriminant functions, using FA percent composition of monovarietal EVOO samples, while Figure 3 shows the plot of the first two discriminant functions, using volatile percent composition.

In the two-dimensional space defined by the first two discriminating functions, the samples belonging to the same cultivar are well discriminated, even if the Dolce Agogia and Moraiolo groups are better concentrated around the centroid of the group. However, the results of the classification show that the samples of all groups are correctly classified.

## 4. Conclusions

The results suggest that total and intrapositional compositions, useful data to characterize the chemical and nutritional properties of the TAG fraction, were able to discriminate monovarietal EVOO samples. The data obtained in this work confirm that genetic factors strongly influence volatile formation and that volatile compounds have a stronger discriminant capacity in respect to FA compositions of TAG fraction. The results showed that the considered statistical approaches permitted discrimination among different cultivars (Dolce Agogia, Frantoio, Leccino, and Moraiolo); in fact, the LDA elaborations were completely significant for the differentiation/classification of the samples. For a practical application of this method, as the classification of EVOO samples of unknown origin, a wider sampling would probably be necessary.

## Figures and Tables

**Table 1:** Total fatty acid (FA) percent composition of triacylglycerol (TAG) fraction of monovarietal extra virgin olive oil (EVOO) samples (% mol, mean values ± SD, *n* = 3).

FA	TAG			
	Dolce Agogia	Frantoio	Leccino	Moraiolo
C16:0	12.3 ± 0.6	12.4 ± 0.8	13.2 ± 0.2	13.1 ± 0.8
C16:1 (*n*-9 + *n*-7)	0.9 ± 0.0	0.9 ± 0.1	0.9 ± 0.1	0.8 ± 0.0
C18:0	2.1 ± 0.1	1.8 ± 0.4	1.9 ± 0.5	1.8 ± 0.2
C18:1 (*n*-9 + *n*-7)	78.0 ± 1.9	77.2 ± 2.0	76.2 ± 2.2	76.5 ± 2.0
C18:2 *n*-6	6.0 ± 0.7	7.1 ± 0.9	7.0 ± 0.8	6.9 ± 1.1
C18:3 *n*-3	0.6 ± 0.0	0.6 ± 0.1	0.8 ± 0.1	0.9 ± 0.2

**Table 2:** Intrapositional FA percent composition of TAG fraction of monovarietal EVOO samples (% mol, mean values ± SD, *n* = 3).

FA	Dolce Agogia	Frantoio	Leccino	Moraiolo
	*sn*-1			
C16:0	15.4 ± 1.9	18.6 ± 1.7	17.5 ± 1.2	17.6 ± 1.6
C16:1 (*n*-9 + *n*-7)	1.1 ± 0.3	1.2 ± 0.5	1.2 ± 0.2	1.0 ± 0.2
C18:0	3.2 ± 0.5	2.8 ± 0.6	3.3 ± 0.7	3.0 ± 0.6
C18:1 (*n*-9 + *n*-7)	73.1 ± 2.9	69.2 ± 2.9	69.4 ± 2.9	70.0 ± 2.4
C18:2 *n*-6	6.7 ± 0.6	7.7 ± 0.7	8.0 ± 0.5	7.8 ± 0.7
C18:3 *n*-3	0.5 ± 0.0	0.5 ± 0.2	0.6 ± 0.0	0.6 ± 0.1
	*sn*-2			
C16:0	0.4 ± 0.1	0.6 ± 0.1	0.5 ± 0.1	0.6 ± 0.1
C16:1 (*n*-9 + *n*-7)	0.5 ± 0.1	0.6 ± 0.1	0.7 ± 0.1	0.7 ± 0.1
C18:0	-	0.1 ± 0.0	0.1 ± 0.0	-
C18:1 (*n*-9 + *n*-7)	89.1 ± 2.3	87.2 ± 2.1	86.8 ± 2.4	87.8 ± 1.9
C18:2 *n*-6	8.9 ± 1.5	10.5 ± 1.8	10.8 ± 2.0	10.0 ± 1.6
C18:3 *n*-3	1.1 ± 0.2	1.0 ± 0.1	1.1 ± 0.0	0.9 ± 0.1
	*sn*-3			
C16:0	21.2 ± 2.6	17.9 ± 3.0	21.7 ± 1.1	20.7 ± 2.5
C16:1 (*n*-9 + *n*-7)	1.2 ± 0.5	0.5 ± 0.2	0.9 ± 0.3	0.9 ± 0.2
C18:0	3.1 ± 0.8	2.9 ± 1.3	2.8 ± 1.8	3.0 ± 0.7
C18:1 (*n*-9 + *n*-7)	71.8 ± 3.1	74.9 ± 4.8	71.9 ± 2.4	71.5 ± 2.7
C18:2 *n*-6	2.1 ± 0.3	3.2 ± 0.7	2.3 ± 1.0	3.0 ± 1.0
C18:3 *n*-3	0.6 ± 0.1	0.6 ± 0.3	0.4 ± 0.1	0.9 ± 0.5

-, not detected.

**Table 3:** Volatile composition of monovarietal EVOO samples (% areas, mean values ± SD, *n* = 3).

Number	Compound	Dolce Agogia	Frantoio	Leccino	Moraiolo
1	ethanol	0.43 ± 0.03	0.61 ± 0.04	0.98 ± 0.28	4.35 ± 0.45
2	pentanal	0.90 ± 0.14	0.68 ± 0.05	0.85 ± 0.14	2.75 ± 0.45
3	*n*-decane	0.81 ± 0.06	0.01 ± 0.01	-	0.98 ± 0.11
4	3-ethyl-1,5-octadiene	0.81 ± 0.07	2.17 ± 0.21	4.02 ± 0.45	1.92 ± 0.28
5	1-penten-3-one	2.18 ± 0.15	5.33 ± 0.27	4.01 ± 0.45	2.74 ± 0.45
6	decadiene	0.53 ± 0.03	1.09 ± 0.15	0.92 ± 0.16	1.07 ± 0.12
7	hexanal	3.01 ± 0.04	4.82 ± 0.30	3.74 ± 0.12	4.55 ± 0.37
8	*trans*-2-pentenal	0.30 ± 0.03	0.37 ± 0.04	0.31 ± 0.03	0.57 ± 0.09
9	*cis*-2-hexenal	0.71 ± 0.02	0.68 ± 0.11	0.80 ± 0.07	0.78 ± 0.12
10	*trans*-2-hexenal	74.45 ± 1.31	79.47 ± 1.52	75.74 ± 1.35	66.07 ± 1.48
11	*o*-cymene	0.40 ± 0.03	0.01 ± 0.00	-	0.10 ± 0.05
12	*n*-hexyl acetate	0.15 ± 0.01	0.34 ± 0.02	0.12 ± 0.02	1.10 ± 0.02
13	*cis*-2-pentenol	1.02 ± 0.03	1.84 ± 0.04	1.07 ± 0.06	1.19 ± 0.08
14	2-heptenal	0.10 ± 0.01	0.20 ± 0.01	0.08 ± 0.01	0.25 ± 0.03
15	1-hexanol	3.69 ± 0.07	0.86 ± 0.05	2.45 ± 0.32	4.58 ± 0.22
16	*cis*-3-hexen-1-ol	0.09 ± 0.01	0.03 ± 0.01	0.04 ± 0.01	0.23 ± 0.00
17	*trans*-3-hexen-1-ol	0.79 ± 0.02	0.98 ± 0.05	1.32 ± 0.72	1.54 ± 0.43
18	*trans*-2-hexen-1-ol	9.05 ± 0.01	1.48 ± 0.16	3.69 ± 0.68	11.98 ± 0.87
19	2,4-hexadienal	1.89 ± 0.09	0.34 ± 0.50	1.68 ± 0.97	0.35 ± 0.49

-, not detected.

**Table 4:** Fisher’s linear discriminant functions and functions at group centroids obtained from LDA analysis using FA or volatiles percent compositions of monovarietal EVOO samples.

Function	FA			Volatiles		
	1	2	3	1	2	3
Eigenvalue	13.773	9.214	2.120	22302.442	435.448	59.557
% of variance	54.8	36.7	8.4	97.8	1.9	0.3
Cumulative (%)	54.8	91.5	100	97.8	99.7	100.0
Canonical correlation	0.965	0.950	0.824	1.000	0.999	0.992
Test of function	1–3	2–3	3	1–3	2–3	3
Wilk’s lambda	0.002	0.031	0.0320	0.000	0.000	0.017
Chi-square	46.159	25.963	8.535	80.779	40.729	16.414
df	33	20	9	18	10	4
Signif.	0.064	0.167	0.481	0.000	0.000	0.003

**Table 5:** Standardized canonical discriminant function coefficients obtained from LDA analysis using total and positional TAG acidic compositions of monovarietal EVOO samples.

**Variable of FA**	**Function**		
	**1**	**2**	**3**
C16:0t	7.80845	2.462617339	1.78735183
C16:1t	−5.0273175	2.482626305	1.12018377
C18:0t	2.9099661	1.892928834	0.80540848
C18:1t	16.271798	8.045815225	4.48269249
C18:2t	13.561999	12.83014703	9.61389748
C18:3t	−0.4295152	0.498737036	1.051572
C16:1 *sn*-1	2.2615419	−0.769331234	−1.38994605
C18:2 *sn*-1	−1.4760586	-1.070614593	−1.77232348
C18:3 *sn*-1	−0.1162137	−2.22366257	0.19232926
C18:1 *sn*-2	0.1016169	9.43991337	5.33667001
C18:3 *sn*-2	0.2085916	−1.495984501	0.82082452
**Variable of volatiles**	**1**	**2**	**3**
ethanol	23.335	−1.046	−0.192
pentanal	−1.182	1.107	0.918
*n*-decane	0.741	1.864	0.847
3-ethyl-1,5-octadien	−18.622	−0.787	−1.674
1-penten-3-one	10.969	−0.711	1.736
*trans*-2-pentenal	0.404	1.228	−1.263

t, total FA% content in TAG fraction; *sn*-1, FA percent content in *sn*-1 position of TAG fraction; *sn*-2, FA percent content in *sn*-2 position of TAG fraction.
